# Associations of Congenital Heart Disease with Deprivation Index by Rural-Urban Maternal Residence: A Population-Based Retrospective Cohort Study in Ontario, Canada

**DOI:** 10.23889/ijpds.v7i3.1772

**Published:** 2022-08-25

**Authors:** Qun Miao, Sandra Dunn, Shi-Wu Wen, Jane Lougheed, Fayza Sharif, Mark Walker

**Affiliations:** 1 BORN Ontario; 2 University of the Witwatersrand; 4 CHEO; 4 University of Ottawa

## Background

The risk of congenital heart disease (CHD) has been found to vary by maternal socioeconomic status (SES) and rural-urban residence. In this study, we examined associations of CHD with two maternal SES indicators and maternal rural-urban residence.

## Approach

This was a population-based retrospective cohort study, including singleton stillbirths and live hospital births from April 1, 2012 to March 31, 2018 in Ontario, Canada. We linked the BORN data and Canadian Institute for Health Information databases. Multivariable logistic regression models were used to examine associations of CHD with material deprivation index (MDI), social deprivation index (SDI), and maternal residence while adjusting for maternal age at birth, parity, assisted reproductive technology, obesity, previous caesarean section, pre-existing health conditions, substance use during pregnancy, and infant’s sex. MDI and SDI were estimated at a dissemination area level and categorized into quintiles (Q1-Q5).

## Results

This cohort included 798,173 singletons. In maternal urban residence, the p trend (Cochran–Armitage test) was < 0.0001 for both MDI and SDI; while for rural residence, it was 0.002 and 0.98, respectively. Infants living in the most materially deprived neighbourhoods (Q5) was associated with higher odd of CHD (aOR: 1.21, 95% CI: 1.12-1.29) compared to Q1. Similarly, infants living in the most socially deprived neighbourhoods (SDI Q5) had 18% increase in the odds of CHD (aOR: 1.18, 95% CI: 1.1-1.26) compared to Q1. Rural infants had 13% increase in the odds of CHD compared to their urban counterparts. After stratifying by maternal rural-urban residence, we still detected higher odds of CHD with two indices in urban residence but only MDI in rural residence.

## Conclusion

Higher material and social deprivation and rural residence were associated with higher odds of CHD. Health interventions and policies should reinforce the need for optimal care for all families, particularly underprivileged families in both rural and urban regions.

